# Role of Allergen Source-Derived Proteases in Sensitization via Airway Epithelial Cells

**DOI:** 10.1155/2012/903659

**Published:** 2012-02-27

**Authors:** Yasuhiro Matsumura

**Affiliations:** Department of Internal Medicine, Akishima Hospital, 1260 Nakagami-Cho, Akishima-Shi, Tokyo 196-0022, Japan

## Abstract

Protease activity is a characteristic common to many allergens. Allergen source-derived proteases interact with lung epithelial cells, which are now thought to play vital roles in both innate and adaptive immune responses. Allergen source-derived proteases act on airway epithelial cells to induce disruption of the tight junctions between epithelial cells, activation of protease-activated receptor-2, and the production of thymic stromal lymphopoietin. These facilitate allergen delivery across epithelial layers and enhance allergenicity or directly activate the immune system through a nonallergic mechanism. Furthermore, they cleave regulatory cell surface molecules involved in allergic reactions. Thus, allergen source-derived proteases are a potentially critical factor in the development of allergic sensitization and appear to be strongly associated with heightened allergenicity.

## 1. Introduction

Asthma is regarded as an inflammatory disorder of the airways and has generally been recognized as being driven by T helper 2- (Th2-) skewed Th cell differentiation. Th2-driven cytokines, interleukin (IL)-4 and IL-13, trigger B cells to synthesize IgE, while IL-5 plays a role in eosinophil maturation and survival, and IL-13 regulates airway hyperresponsiveness and mucus hyperplasia.

Epithelial cells clearly play important roles in the initiation of Th2 cell responses to allergens. The epithelial cell layer also acts as a molecular sieve that excludes invaders and plays an important role in homeostasis. Barrier function disorder due to filaggrin (FLG) mutations is critical in the pathogenesis of atopic dermatitis [1]. Although FLG is not expressed in the lower airway respiratory epithelium [2], barrier function of the airway epithelium is impaired in asthma, showing shared common underlying pathogenic mechanisms.

Taking these findings together, asthma can be viewed as a disease of both excessive activation and impairment of airway epithelial barrier function [3–5].

Sources of allergens, such as pollen, house dust mites (HDMs), cockroaches, and fungi, may produce or contain proteases and thereby activate and disrupt the epithelial barrier, causing greater sensitization.

This paper focuses on the importance of allergen source-derived proteases as a factor contributing to primary sensitization to allergens and to exacerbation of allergic disorders secondary to impaired epithelial barrier function.

## 2. Allergen Source-Derived Proteases

Environmental exposure to allergens is an important determinant of the prevalence of asthma. Allergen source-derived proteases act not only as allergens, but also as promoters of allergenicity.

### 2.1. Pollens

Pollen allergens have protease activity. The pollens of Japanese cedar (*Cryptomeria japonica*), Japanese cypress (*Chamaecyparis obtusa*), and Rocky Mountain juniper (*Juniperus scopulorum*) contain serine protease activity [6]. An aspartic protease was also recently identified in Japanese cedar pollen allergen [7]. In grass, two serine proteases from short ragweed (*Ambrosia artemisiifolia*) pollen have been purified and characterized [8, 9]. Kentucky blue grass (*Poa pratensis*), rye grass (*Lolium perenne*), and Bermuda grass (*Cynodon dactylon*), pollen have also been characterized. These pollens exhibited peptidase activity, which appeared to be from serine proteases, but cystein protease activity was also detected in Kentucky and rye grass pollen [10]. Grass pollen major group 1 allergens are reported to be cysteine proteases [11, 12]. The pollens of white birch (*Betula alba*) and short ragweed contain not only serine but also cysteine protease activity [6, 13].

### 2.2. HDMs

HDMs produce cysteine and serine proteases. *Dermatophagoides pteronyssinus* 1 (Der p 1) and Der p 3 [14] are cysteine proteases. Der p 6 and Der p 9 are serine proteases [15–17]. Interestingly, Der p 2, which lacks apparent protease activity, is a structural mimic of MD2, a component of the Toll-like receptor-4 (TLR-4) complex, and can reconstitute a TLR4 signaling complex [18], independently of protease effects.

### 2.3. Cockroaches

American cockroach (*Periplaneta americana*) and German cockroach (*Blattella germanica*) allergen extracts have complex proteolytic activities [19–21]. An approximately 28 kDa trypsin-like serine protease (Per a 10) was purified and characterized from the whole body extract of American cockroaches [22, 23]. Bla g 2, a potent allergen from German cockroaches, has been identified as an aspartic protease [24, 25]. German cockroach extract is rich in proteases and exerts direct proinflammatory effects on airway epithelial cells. These proinflammatory effects are abolished by serine inhibitors [26], suggesting the involvement of a serine protease. However, the presence and activities of proteases in cockroach extracts, especially those targeting aspartate, cysteine, and serine, remain controversial [27].

### 2.4. Fungi

A large number of mold species are known to harbor proteases. Serine proteases of airborne fungi have been identified in *Penicillium*, *Aspergillus*, *Rhodotorula*, *Curvularia*, and *Cladosporium *species [28–31]. Cross-reactivity has been reported among fungal species [32–35]. The active protease of *Epicoccum purpurascens*, Epi p 1, which is a potent fungal allergen source inducing respiratory allergic disorders worldwide, also plays an important role in driving allergic responses in the airways of murine models [36].

Recent research has focused on the role of exogenous allergen proteases in allergic disorders. Enzymatic activities have been proposed to facilitate sensitization to various allergens [37–39].

## 3. Disruption of Epithelial-Cell Barrier

In the clinical setting of asthma, there is evidence that the barrier function of the airway epithelium is impaired [40–43]. The airway epithelium serves as a barrier via the formation of tight junctions (TJs) which seal off the paracellular space. TJs also have gate functions that regulate the passage of ions and macromolecules through the paracellular pathway. TJs are comprised of a series of interacting proteins and receptors including zonula occludens (ZOs) proteins ZO-1–3, occludin, claudins 1–5, and transmembrane adhesion proteins (*β*-catenin, E-cadherin, and junctional adhesion molecule-1). These proteins and receptors appear to interact in a homophilic manner. ZO-1, -2, and -3 bind to the cytoplasmic tail of occludin and link the TJ to the actin cytoskeleton. Occludin appears to copolymerize to form claudin-based TJ strands. Claudins adhere to each other in a homotypic as well as a heterotypic manner, determining the barrier properties of cell-cell contact existing between two neighboring cells, and regulate paracellular permeability. Regulatory molecules, including tyrosine kinases, proteases, and GTPases, colocalize near the tight junction. Coordinated functions between the transmembrane components and cytoplasmic molecules, along with the cytoskeleton and regulatory molecules, play a crucial role in not only barrier function but also communication between adjacent cells as well as in the regulation of intercellular transport [44, 45].

Initiation of sensitization to allergens in the airway is preceded by their uptake and processing by a subpopulation of mucosal dendritic cells (DCs), followed by presentation of specific peptide epitopes to naïve T cells in association with major histocompatibility (MHC) class II. Mucosal DCs are positioned within the epithelium. DCs extend their processes between epithelial cells directly into the airway lumen, as a periscope function that allows continuous immune surveillance of the airway luminal surface. DCs form TJs with epithelial cells through their expression of adhesion molecules and via E-cadherin homotypic interactions [46, 47].

DCs act as immune sentinels by alerting T cells to the presence of antigens after delivery and presentation to draining lymph nodes. In mice, antigen administered into the lungs is rapidly, that is, in as little as 12 hours, transported to thoracic lymph nodes [48, 49]. The path taken by inhaled antigens from the airways to sampling by DC subsets has yet to be characterized in detail. Antigen sampling functions may also differ between DCs located in the alveolar wall and mucosal DCs that line the conducting airways [47, 50–52].

Although the sampling function of airway DCs ensures that any inhaled protein will be recognized and presented to T cells, allergen source-derived proteases compromise epithelial barrier function by degrading TJ proteins, thus facilitating allergen delivery across epithelial layers.

Proteases released by major allergenic pollens have been shown to injure airway epithelial cells *in vitro *[53]. Proteolytic enzymes contained in pollens of giant ragweed (*Ambrosia trifida*), white birch, Kentucky blue grass, and Easter lily (*Lilium longiflorum*) facilitate allergen delivery across epithelia by degrading occludin, resulting in disruption of epithelial TJs. This effect was blocked by inhibitors of serine and cysteine proteases in Madin-Darby canine kidney (MDCK) and Calu-3 cells [54].

Der p 1 increased epithelial permeability by disrupting TJs [55]. Immunoblotting demonstrated that the disruption of TJ morphology by Der p 1 was associated with cleavage of ZO-1 and occludin in MDCK and 16HBE14o-human bronchial epithelial cell lines [56]. Putative Der p 1 cleavage sites were found in peptides from an extracellular domain of occludin and in the TJ adhesion protein claudin-1. Extracellular cleavage of TJs initiates intracellular processing of junctional constituents. Der p 1 is also envisaged to operate indirectly on TJs by activating a cell surface zymogen, which then proceeds to cleave TJs [57]. ZO-1 is intracellular and is therefore unlikely to be directly degraded by Der p 1, and its breakdown is presumed to be a consequence of TJ disassembly [57].

Der p 1 and *Dermatophagoides farinae* 1 (Der f 1) can inactivate lung surfactant proteins (SP)-A and -D [58], which are predominantly synthesized and secreted in the lung by alveolar type II cells and Clara cells. SP-A and-D are known to play not only significant roles in innate immune defense such as bacterial aggregation and modulation of leukocyte function, but also are implicated in the allergic response [59, 60].

Allergens, derived from cockroach extracts, are reported to increase the permeability of bronchial airway epithelial cells indirectly through the induction of vascular endothelial growth factor [61] and thereby gain access to intraepithelial DCs. 

*Aspergillus fumigatus* proteinase directly induces human epithelial cell detachment [62]. Pen ch 13, a major allergen of *Penicillium chrysogenum,* is a serine protease. Its enzymatic activity damages the epithelial barrier by cleaving the TJ protein occludin at Gln202 and Gln211, amino acids within the second extracellular domain of the protein on 16HBE14o-cells [63], followed by the induction of proinflammatory responses in epithelial cells.

Epithelial injury and aberrant repair are involved in triggering asthma. Interestingly, Pen ch 13 decreases cell surface expression of CD44 in 16HBE14o-cells and primary bronchial epithelial cells [64], which has been suggested to contribute to repair of epithelial damage [65]. CD44 is a transmembrane adhesion molecule and the major receptor for hyaluronan, a major extracellular matrix component. CD44 is important for the removal of extracellular matrix from sites of tissue injury, and impaired clearance of hyaluronan results in persistent inflammation [66, 67].

 Thus, loss of epithelial barrier function, as a consequence of proteases associated with allergens, facilitates antigen access to DCs. The result is that the adaptive immune response is skewed towards Th2 cells, and the IgE immune response is amplified.

 Most results are based on in vitro study. Since the digestion process of proteases needs a suffientcient local concentration and time, dilution in mucus, as well mucociliary clearance of the respiratory tract, may complicate the digestion process of TJs in vivo.

## 4. Allergen Source-Derived Proteases Activate Pattern Recognition Receptors (PRRs)

Pulmonary epithelial cells are now thought to play vital roles in both innate and adaptive immune responses. Epithelial cells can sense and respond to inhaled allergens or proteases via activation of a variety of pattern recognition receptors (PRRs) such as TLR and PAR. These activated receptor signals trigger nuclear factor *κ*B (NF-*κ*B) activation, leading to transcriptional activation of several proinflammatory genes including those encoding cytokines and chemokines. Epithelial production of thymic stromal lymphopoietin (TSLP), granulocyte-macrophage colony stimulating factor (GM-CSF), and IL-33 and IL-25, as well as the production of chemokines, both attract and activate DCs, skewing T-cell production toward to the Th2 subset.

## 5. PAR-2

Protease allergens are reported to elicit non-IgE-mediated airway reactions by triggering innate immunity receptors, such as PARs, to activate epithelial cells, mast cells, and DCs, which in turn leads to further release of mediators [68]. PARs constitute a novel family of seven-transmembrane G-protein-coupled receptors. To date, four PARs have been identified and cloned. They are widely expressed on cells comprising blood vessels, connective tissue, epithelium, and airways, as well as on leukocytes [69]. PARs are activated by proteolytic cleavage at the amino terminus, allowing interaction between the newly formed “tethered ligand” and the second extracellular loop of the receptor. This interaction confers a cellular signaling property. PARs can also be activated by small peptides that mimic the tethered ligand. Activated PARs coupled to G-signaling cascades increase phospholipase C level, which in turn raises intracellular calcium (Ca^2+^) level [70–72]. G protein activation also generates a transcriptional response through extracellular signal-regulated and mitogen-activated protein kinases, as well as NF-*κ*B [73–75].

In patients with bronchial asthma, PAR-2 expression is increased on the surface of respiratory epithelial cells [76, 77]. PAR2 agonists induce constriction of human bronchi [78]. Lack of PAR-2 expression is reported to lower inflammatory cell infiltration and reduce airway hyperreactivity in response to allergen challenge in mice [79]. 

Asthma is associated with increased water and chloride (Cl^−^) secretion into the airway lumen due to elevated expression of Ca^2+^-activated Cl^−^ channels [80–82]. Stimulation of PAR-2 receptors in mouse and human airways inhibited amiloride-sensitive sodium (Na^+^) conductance and stimulated luminal Cl^−^ channels and basolateral potassium (K^+^) channels, which together may cause accumulation of airway surface fluid [83].

Activation of PAR-2 was, however, shown to reduce airway inflammation in a rabbit model of experimental asthma [84], which supports the concept of PAR-2 being a cytoprotective receptor involved in prostanoid-dependent cytoprotection in the airways. Prostaglandin E (PGE), which inhibits pulmonary infiltration by immune cells and bronchial constriction in allergen-induced asthma, is produced by cultured airway smooth muscle cells [85, 86], as well as airway epithelial cells, follicular DCs, fibroblasts, monocytes, and alveolar macrophages [87]. Intranasal administration of PAR-2-AP was shown to inhibit airway eosinophilia and hyperresponsiveness in allergic mice via cyclooxygenase- (COX-) 2-dependent generation of PGE_2_ [88].

Thus, whether the activation of PAR-2 promotes or opposes the progression of airway inflammatory responses depends on the experimental model and species and is not yet fully understood. This is an area requiring further research.

PAR-2 is a major candidate for sensing environmental exposure to serine proteases. PAR-2 is involved in antigen-induced asthmatic responses, including increase in IgE production, a heightened methacholine response, upregulated production of IL-6, IL-8, GM-CSF, and eotaxin, increased matrix metalloproteinase-9 (MMP-9) release, and relaxation of bronchi [89, 90]. Interestingly, MMP-9, which plays an important role in remodeling of the airways in disease, is hypothesized to exert its effects on the epithelium by cleaving one or more components of cell-cell junctions and triggering anoikis [91].

Increased release of proinflammatory cytokines, such as IL-6 and IL-8, from airway epithelial cells in response to proteases contained HDM [92, 93]. Der p 3 and Der p 9 may induce a non-allergic inflammatory response in the airways, via release of proinflammatory cytokines from the bronchial epithelium, which is at least partially mediated by PAR-2 [94]. Although release of IL-6 and IL-8, due to the protease activity of Der p 1, can occur via a mechanism independent of Ca^2+^ mobilization and PAR activation [95, 96], their release from an A549 cell line was reported to be associated with PAR-2 [97].

Fungal proteases, from *Aspergillus fumigates*, *Alternaria alternate,* and *Cladosporium herbarum*, differentially induced morphologic changes, cell desquamation, and the production of various cytokines [98, 99]. The protease activity of Pen ch 13, an allergen from *Penicillium chrysogenum*, is required for the induction of PGE2, IL-8, transforming-growth-factor- (TGF-) beta1 and COX-2 expression in A549 cells, 16HBE14o-cells, and primary cultures of HBEpC [63]. Pen c 13, the main allergen produced by *Penicillium citrinum*, induces the expression of IL-8 in human airway epithelial cells by activating either PAR-1 or PAR-2 [100].

Fecal remnants [101] and extracts [102] of German cockroach induced mucosal allergic sensitization and inflammation via PAR-2 in mice. Inflammatory responses of human eosinophils to German and Oriental cockroach (*Blatta orientalis*) extract antigens are mediated via PAR-2 [27, 103]. Recent data provide evidence implicating the protease activity of cockroaches in cytokine regulation. Allergens of German and American cockroaches induce IL-8 expression in H292 cells [104] and A549 cells [105], respectively, and both are blocked by serine protease inhibitors, suggesting PAR-2 might play a role in cockroach allergen-induced IL-8 secretion from human airway epithelial cells [105]. Proteases in German cockroach extract regulate PAR-2 and extracellular signal-regulated kinase (ERK) to increase NF for IL-6 (NF-IL6) activity (recently known as C/EBP-*β*), as well as synergistically regulating TNF-*α*-induced IL-8 promoter activity in the human airway epithelium [104, 106–108]. German cockroach fecal remnants contain active serine proteases, which augment TNF-*α*-induced MMP-9 expression via a mechanism that involves PAR-2, ERK, and AP-1 [109].

Interestingly, exposure to inhaled antigens with a PAR-2-activating peptide led to allergic sensitization, whereas exposure to Ag alone induced tolerance in BALB/c mice administered ovalbumin (OVA), suggesting PAR-2 activation in the airways at the time of inhaled antigen exposure to be capable of shifting the resulting immune response toward allergic sensitization and the development of asthma. Furthermore, PAR-2-mediated allergic sensitization is reported to be TNF dependent [110].

## 6. Thymic Stromal Lymphopoietin

In addition to serving as a physical barrier, airway epithelial cells are now thought to play essential roles in allergic responses. TSLP [111] is expressed mainly by epithelial cells comprising the barrier surfaces of the lungs. Genetic analyses of atopic populations have demonstrated polymorphisms in TSLP to be associated with asthma and airway hyperresponsiveness, IgE concentration, and eosinophilia [112–116]. Overexpression of TSLP in the lungs can trigger Th2 cell immunity. Mice expressing TSLP in the lungs spontaneously develop an airway inflammatory disorder with characteristics similar to those of human asthma [117].

Studies of endobronchial biopsy specimens and BAL fluid of subjects with severe asthma have shown that asthma is associated with elevated bronchial mucosal expression of TSLP and Th1-attracting (IP-10/CXCL10) and Th2-attracting (TARC/CCL17, MDC/CCL22) chemokines [118, 119].

**Figure 1:**
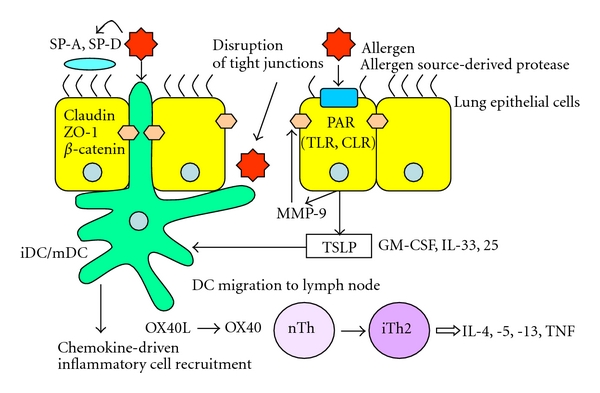
Allergen source-derived proteases compromise epithelial barrier function by degrading TJ proteins, facilitating allergen accessibility to DCs. Enzymatically active allergens can activate PAR to induce TSLP. TSLP induces immediate innate immune functions in DCs, leading to recruitment of inflammatory cells. TSLP triggers the maturation of DCs, so they migrate to mediastinal lymph nodes. Induction of DCs to upregulate OX40L by TSLP promotes Th2 responses. PAR also upregulates production of MMP-9, which degrades tight junction proteins. Thus, impairment of airway epithelial barrier function and activation of epithelial cells are involved in the pathogenesis of inflammation mediated by allergen source-derived proteases.

IL-25 and TSLP perform important functions in the initiation of allergic responses [120–122]. TSLP expression is induced in airway epithelial cells by exposure to allergen-derived proteases, and PAR-2 is involved in this process. A recent study demonstrated upregulation of IL-25 and TSLP mRNA in pulmonary epithelial cells after protease allergen treatment *in vivo* and *in vitro*, and that the induction of IL-25 and TSLP occurs via the intracellular ERK and p38 MAP kinase pathways [123]. TSLP induces the innate immune functions of DCs, leading to chemokine-driven recruitment of Th2 cells to the airway, and these cells then produce Th2 type cytokines. TSLP also triggers the maturation of DCs and their migration to mediastinal lymph nodes, again skewing the T-cell distribution in favor of inflammatory Th2 cells producing IL-4, IL-5, IL-13, and TNF-*α*. These processes involve interactions between costimulatory molecules, such as OX40 (CD134) in the membranes of naïve T cells and OX40L (CD134L) in the membranes of DCs [124, 125]. TSLP was reported to be induced in the airway epithelial cell line BEAS-2B by exposure to *Alternaria* proteases [126] (see Figure 1).

Basophils are directly activated by protease allergens and produce TSLP. Cysteine protease activity of papain, an occupational allergen homologous to Der f 1 and Der p 1, was reported to initiate Th2 sensitization *in vivo* in mice via activation of basophils [122].

## 7. Allergen Source-Derived Proteases Enhance Sensitization to Other Allergens and Allergen Components

The tertiary structure of an allergen is involved in IgE-binding activity. The tertiary architecture of the Der p 1 molecule itself is not sufficient to induce major production of both IgE and IgG, but its proteolytic activity is crucial for eliciting positive immune responses in naïve mice [127].

Immunization of mice with proteolytically active Der p 1 results in significant increases in total IgE and Der p 1-specific IgE synthesis, as compared with animals immunized with Der p 1 irreversibly blocked with E-64, a cysteine protease inhibitor [128]. The proteolytic activity of Der p 1 heightens inflammatory cell infiltration into the lungs and systemic IgE production when administered directly into the respiratory tract [129].

Allergens with protease activity are also able to mediate sensitization to nonprotease proteins. Exposure to HDM extract establishes a mucosal environment fostering the development of allergic sensitization to otherwise weak or innocuous antigens, such as OVA, suggesting that whether an airborne allergen will generate allergic airway disease may depend, at least in part, not simply on being exposed to it but rather the setting in which that exposure takes place [130, 131].

Active protease contents of fungal extracts can influence the induction and severity of allergic airway disease in mice. Proteolytically active molecules can facilitate the presentation of nonproteolytic allergens to the immune system, thereby augmenting sensitization to allergens. These proteolytic allergens thereby promote Th2 cell sensitization. For a mature response, the participation of components such as enzymes may have a major role, as is suggested by the response reported with crude antigen and recombinant allergens. Alkaline serine proteases are major allergens of *Aspergillus* species. The alkaline serine protease allergen of *A. fumigatus* (Asp f 13) induces IgE as well as an inflammatory response and has synergistic effects on the Asp f 2-induced immune response in mice [31, 132].

## 8. Allergen Source-Derived Proteases Cleave Cell Surface Molecules: Roles beyond the Airways

Allergen source-derived proteases have been recognized as having the ability to cleave key regulatory molecules in allergic reactions involving cell surfaces, and to amplify IgE responses.

Previously suggested pathogenic roles of exogenous proteases, especially Der p 1, involve cleavage of various endogenous proteins, including removal of low-affinity IgER (CD23) from the surface of human B lymphocytes. This loss of cell surface CD23 from IgE-secreting B cells may promote and amplify IgE immune responses by eliminating an important inhibitory feedback mechanism that would normally limit IgE synthesis. Furthermore, fragments of CD23 released by Der p 1 may directly promote IgE synthesis [133].

Der p 1 cleaves the *α* subunit of the IL-2 receptor (IL-2R or CD25), which is pivotal for Th1 cell propagation, removing it from the surface of human peripheral blood T cells. As a result, these cells show markedly diminished proliferation and interferon *γ* secretion in response to a potent stimulus such as anti-CD3 antibody. IL-2R cleavage by Der p 1 is likely to cause impaired growth of cells of the Th1 subset and may, as a consequence, bias the immune response toward Th2 cells [134, 135].

Der p 1 also cleaves cell surface DC-SIGN and DC-SIGNR, which are closely related C-type lectin transmembrane receptors expressed within compartments of the immune system. These molecules then bind to intracellular adhesion molecule-2 (ICAM-2), expressed on endothelial and T cells, and ICAM-3, expressed on T cells. Both are involved in DC trafficking, DC-T-cell interactions, and skewing of the immune response in favor of Th 1 [136].

Proteolytic activity of Der p 1 results in cleavage of CD40 from the DC surface. This deprives DCs of the ability to receive CD40L-mediated signals from T cells, which is an important pathway stimulating IL-12 production. This downregulation of IL-12 may enable DCs to directly promote the differentiation of naïve T cells toward the Th2 cytokine profile [137].

## 9. Conclusion

Although genetic aspects of airway epithelium barrier deficiency have yet to be determined, both structural and functional abnormalities of the epithelium underlie the pathogenesis of bronchial asthma. Protease activity in allergens confirms that allergenicity not only results from the reaction to an epitope, which is involved in adaptive immune responses by T and B cells, but also from disruption of airway barrier function and activation of innate immune responses through epithelial cells [138–140]. Stimulation of PAR-2 signaling by protease allergens participates in the inflammatory process, and may serve as a link between innate and adaptive immune responses.

Analysis of these allergen proteases, which constitute protease-sensing pathways in airway epithelial cells, is essential for elucidating the pathogenesis of allergic asthma. A full understanding of these processes is anticipated to lead to both treatment and preventive measures against asthma development.
